# Emerging Role of Exosomes in Diagnosis and Treatment of Infectious and Inflammatory Bowel Diseases

**DOI:** 10.3390/cells9051111

**Published:** 2020-04-30

**Authors:** Anaïs Larabi, Nicolas Barnich, Hang Thi Thu Nguyen

**Affiliations:** M2iSH, UMR 1071 Inserm, Université Clermont Auvergne, INRAE USC 2018, CRNH, 63001 Clermont-Ferrand, France; anais.larabi@gmail.com (A.L.); nicolas.barnich@uca.fr (N.B.)

**Keywords:** exosomes, bacterial infection, immune response, inflammatory bowel disease, vaccine

## Abstract

To communicate with each other, cells release exosomes that transfer their composition, including lipids, proteins and nucleic acids, to neighboring cells, thus playing a role in various pathophysiological processes. During an infection with pathogenic bacteria, such as adherent-invasive *E. coli* (AIEC) associated with Crohn disease, exosomes secreted by infected cells can have an impact on the innate immune responses of surrounding cells to infection. Furthermore, inflammation can be amplified via the exosomal shuttle during infection with pathogenic bacteria, which could contribute to the development of the associated disease. Since these vesicles can be released in various biological fluids, changes in exosomal content may provide a means for the identification of non-invasive biomarkers for infectious and inflammatory bowel diseases. Moreover, evidence suggests that exosomes could be used as vaccines to prime the immune system to recognize and kill invading pathogens, and as therapeutic components relieving intestinal inflammation. Here, we summarize the current knowledge on the role of exosomes in bacterial infections and highlight their potential use as biomarkers, vaccines and conveyers of therapeutic molecules in inflammatory bowel diseases.

## 1. Introduction

Cell-to-cell communication is essential to maintain homeostasis in a multicellular organism. Exosomes, small extracellular vesicles of 30 to 100 nm produced by most cell types and delivered into bodily fluids, are a part of a larger network by which cells communicate with each other [1]. These vesicles arise from a unique biogenesis pathway starting with the inward budding of the plasma membrane to form membrane-enclosed compartment called early endosomes [2]. These early endosomes then mature into late endosomes that accumulate intraluminal vesicles (ILVs) in their lumen. These ILVs are formed by inward budding of the membrane of early endosomes, a process mediated by the endosomal sorting complex required for transport (ESCRT) or by ESCRT-independent mechanisms involving tetraspanins or lipids such as lysobisphosphatidic acid and ceramides [2]. During their formation, ILVs are loaded with biologically active molecules, including lipids [3], proteins [4] and nucleic acids [5]. Late endosomes then fuse either with lysosomes, leading to the degradation of their content, or with the plasma membrane to release ILVs into the extracellular space, generating vesicles referred to as “exosomes” [2].

Although initially considered as cellular waste [6], new conceptions supported by recent data propose that exosomes are an alternative way to eliminate waste products and maintain cellular homeostasis [7], and that these vesicles play a role in transmitting information and in activating biological responses in nearby or distant target cells [8]. Indeed, secreted exosomes may elicit cellular responses via different mechanisms: (i) soluble and juxtacrine signaling that involve the direct stimulation of target cell receptors by cleaved or membrane-bound exosomal ligands, respectively; (ii) fusion of exosomes with the plasma membrane of the recipient cell and transfer of the exosomal content into the cell cytoplasm; (iii) phagocytosis; (iv) micropinocytosis; and (v) receptor-mediated endocytosis of exosomes by the recipient cell [9], based on clathrin or caveolin depending on recipient cells [10]. Thus, exosomes have been associated with numerous physiological and pathological functions [8]. Under physiological conditions, adipose tissue-derived pro-inflammatory M1 and anti-inflammatory M2 macrophages secrete exosomes that regulate neighboring adipocytes’ sensitivity to insulin [11]. Indeed, M1 macrophages release exosomes that deliver miR-155, which targets peroxisome proliferator-activated receptor gamma (PPARγ) to adipocytes, thus increasing their resistance to insulin, while M2 macrophages release exosomes that increase adipocytes’ sensitivity to insulin [11]. Moreover, adipose tissue-derived stem cells secrete exosomes that carry the transcription factor signal transducer and activator of transcription 2 (STAT2) and target adipose tissue-derived macrophages to promote the conversion or accumulation of M2 macrophages [12]. These M2 macrophages promote adipose tissue-derived stem cells, which facilitate immune and metabolic homeostasis in white adipose tissue [12]. Moreover, exosomes play a role in cellular homeostasis, tissue regeneration and wound repair. Indeed, exosomes and microvesicles derived from human liver stem cells favor hepatic regeneration in rats [13]. Upon injury, epithelial cells secrete a higher number of exosomes that carry transforming growth factor-beta (TGF-β) mRNA, which stimulates fibroblast differentiation to initiate tissue-regenerative responses [14]. Similarly, exosomes released by intestinal epithelial cells contain annexin A1, which participates in epithelial repair [15]. Thus, these vesicles may promote epithelial wound repair [15]. Conversely, osteoclasts, besides regulating bone resorption, release exosomes that can transfer miR-214-3p to osteoblasts, thus inhibiting bone formation [16]. Under pathological conditions, such as inflammation or infection, the release and content of exosomes can be modified, thereby influencing inflammatory responses and immunity [17]. In particular, exosomes seem to play a role in inflammatory bowel diseases (IBD), a family of chronic inflammatory disease of the gastrointestinal tract including Crohn disease (CD) and ulcerative colitis (UC). The etiology of IBD is multifactorial, involving environmental factors, genetic susceptibility and infectious agents, leading to an exacerbated mucosal immune response against intestinal microbiota [18]. Our group has focused on investigating the implication of adherent-invasive *Escherichia coli* (AIEC) in the infectious etiology of CD [19]. We recently showed that upon infection with AIEC, intestinal epithelial cells and immune cells secrete an increased amount of exosomes that are, in turn, uptaken by uninfected cells, leading to an enhanced pro-inflammatory response and defective clearance of intracellular AIEC in the latter [20]. In the context of infection with other pathogens, such as *Salmonella enterica* serovar Typhimurium, *Toxoplasma gondii*, *Mycobacterium tuberculosis* or *Mycobacterium bovis*, it has been reported that exosomes secreted by infected cells contain bacterial antigens [21,22,23], as well as bacteria- and host-derived nucleic acids [24,25,26], and modulate immune responses of uninfected surrounding cells [21,22,23,24,26,27]. Due to their presence and high stability in most bodily fluids as well as the variation in their content in pathological conditions, exosomes have great potential to serve as biomarkers [28]. Moreover, exosomes can package a variety of biomolecules and drugs, making these vesicles potential carriers of therapeutic compounds [28]. This review presents the advanced knowledge of exosome functions in bacterial infection and IBD and highlights their potential role as diagnostic biomarkers and vaccines as well as conveyers of therapeutic molecules.

## 2. Exosomes and Immune Responses: A Focus on Bacterial Infection

### 2.1. Exosomes and Innate Immune Responses

Evidence has shown a role for exosomes in modulating innate immune responses [1]. During bacterial infection, small molecules such as N-formylmethionyl-leucyl-phenylalanine peptides are released and initiate the chemotactic recruitment of neutrophils, which then amplify their recruitment by secreting a secondary chemoattractant, the lipid eicosanoid leukotriene B_4_ (LTB_4_) [29]. It was shown that upon stimulation by the N-formylmethionyl-leucyl-phenylalanine peptides, neutrophils release LTB_4_ and its synthesizing enzymes in exosomes [30]. These exosomes then activate resting neutrophils and elicit chemotactic activity in an LTB_4_-dependent manner [30]. Moreover, exosomes may increase the stability of the LTB_4_ gradient compared with direct secretion and, by their time-delayed secretion of LTB_4_, extend the lifetime of this gradient after the decrease in the primary stimulus [31].

It has been shown both in vitro and in vivo that activation of immune cells by a foreign element such as a pathogen leads to the secretion of exosomes with an altered cargo that can activate immune responses in an antigen presentation-independent or -dependent manner. Indeed, exosomes secreted by dendritic cells stimulated with lipopolysaccharide (LPS) contain soluble tumor necrosis factor alpha (TNF-α) [32]. These exosomes induce the secretion of pro-inflammatory cytokines and chemokines, such as interleukin (IL)-8, TNF-α and monocyte chemoattractant protein (MCP) 1 by epithelial cells in vitro in an exosomal TNF-α-dependent manner [32]. Similarly, upon infection with *Mycoplasma*, dendritic cells secrete exosomes that induce the proliferation of B lymphocytes independently of antigen presentation and without the help of T lymphocytes [33]. The infection of bladder urothelial cells with uropathogenic *E. coli* induces the activation of pyroptosis and the secretion of exosomes containing IL-1β and IL-18 that act as a chemoattractant for mast cells, which then aggravate bladder urothelial barrier dysfunction via the secretion of tryptase [34].

Exosomes also activate immune responses in an antigen-dependent manner. It has been reported that exosomes secreted by macrophages infected with *Mycobacterium tuberculosis*, *M. bovis* BCG or *Salmonella* Typhimurium contain pathogen-associated molecular patterns (PAMPs) [21]. Indeed, some mycobacterial proteins may contain a signal targeting them to ILVs, thus favoring their secretion via exosomes [23]. Analysis of the protein composition of exosomes secreted by macrophages infected with *M. tuberculosis* showed an increase in the abundance of 41 human proteins, among which 63% were predicted to be associated with the exosomal membrane [35]. Moreover, exosomes secreted by macrophages infected with *M. tuberculosis* carry the 19 kDa LpqH lipoprotein [23], which favors inflammation and stimulates macrophage activation and interferon gamma (IFN-γ) expression via the Toll-like receptor (TLR) 2/myeloid differentiation protein (MyD) 88 pathway [36]. Exosomes released by *M. avium*-infected macrophages promote the secretion of the pro-inflammatory cytokines IFN-γ and TNF-α by exosome-receiving macrophages [37]. The increased TNF-α secretion may be due to an increased level of heat shock protein (HSP) 70 in exosomes secreted by *M. avium*-infected macrophages [38]. Both exosomes and HSP70 induce the activation of recipient macrophages, in particular nuclear factor-kappa B (NF-κB) activation and TNF-α release [38]. Similar results were reported for *M. smegmatis* infection [38]. Exosomes secreted by *M. avium*-infected macrophages also contain glycopeptidolipids, which can be transferred to uninfected recipient macrophages, inducing a pro-inflammatory response [39]. However, exosomes secreted by *M. tuberculosis*-infected macrophages can also restrict the activation of recipient macrophages in response to IFN-γ stimulation [36].

In vivo, the intranasal administration of exosomes secreted by murine macrophages infected with *M. tuberculosis* or *M. bovis* to mice increases the secretion of TNF-α and IL-12, the recruitment of neutrophils as well as the activation of dendritic cells and macrophages [21,22,23]. Similarly, the stimulation of murine macrophages with exosomes purified from the bronchoalveolar lavage fluid [21] or the serum [27] of mice infected with *M. bovis* increases the production of pro-inflammatory cytokines by these cells [21]. Finally, macrophages treated with exosomes secreted by *M. tuberculosis*-infected macrophages release significant amounts of chemokines and could promote macrophage recruitment in vivo [27].

Viable intracellular *M. tuberculosis* are also able to produce bacterial membrane vesicles containing *M. tuberculosis* lipoglycans and lipoproteins within host macrophages, which are subsequently released extracellularly [40]. These bacterial vesicles, but not exosomes derived from *M. tuberculosis*-infected macrophages, activate TLR2 signaling and the secretion of IL-8, TNF-α, IL-12p40 and IL-10 cytokines by uninfected recipient macrophages [40]. This suggests that the activation of immune responses in uninfected exosome-receiving macrophages is mostly due to the release of *M. tuberculosis* vesicles rather than host cell-derived exosomes into extracellular milieu [40]. Thus, further studies are needed to decipher the specific role of bacterial and host vesicles in the context of bacterial infection.

Exosomes secreted by macrophages infected with mycobacteria also carry functional bacterial RNAs as well as host cell mRNAs [24] and miRNAs that may target various metabolic pathways of the host following infection [25,41]. Indeed, upon *M. tuberculosis* infection, macrophages as well as exosomes secreted by these cells exhibit an increase in the level of miR-18a [42]. This miRNA favors *M. tuberculosis* survival in infected macrophages by counteracting autophagy, however, the impact of miR-18a released into exosomes from infected macrophages on *M. tuberculosis* survival in exosome-receiving cells has yet to be analyzed [42]. Similarly, the infection of human macrophages with *Yersinia pestis* and *Bacillus anthracis* leads to a variation in exosomal miRNA cargo in a pathogen-dependent manner [43], suggesting that the secretion of miRNAs in exosomes may play a role in infection. After LPS exposure, murine bone marrow-derived dendritic cells (BMDC) secrete exosomes containing miR-146a and miR-155. These miRNAs are efficiently transferred to recipient cells in which they modulate inflammatory gene expression and cell responses to endotoxins [44]. Similarly, exosomes derived from the serum of mice that exhibit acute sepsis-related lung injury induced by intraperitoneal LPS injection are selectively enriched in pro-inflammatory miR-155 [45]. These exosomes induce NF-κB activation and the production of TNF-α and IL-6 and promote proliferation of macrophages in vitro [45]. In vivo, the intravenous injection of these exosomes to naïve mice increases the recruitment of pro-inflammatory M1 macrophages to the lungs and induces lung inflammation [45]. Exosomes from macrophages infected with *Helicobacter pylori* also exhibit an increase in miR-155 level, which exacerbates inflammatory responses in recipient macrophages, thus limiting *H. pylori* replication and preventing *H. pylori*-induced gastritis [46].

Exosomes also play a role in the dissemination of bacterial virulence factors. Indeed, the major virulence factor cytotoxin-associated gene A (CagA) secreted by *H. pylori* was found in exosomes from gastric epithelial cells inducibly expressing the *cagA* gene as well as in exosomes from patients infected with a CagA-positive *H. pylori* strain. These exosomes may pass into systemic circulation and deliver CagA to tissues and organs nearby or at a distance from the site of infection [47]. At the systemic level in individuals infected with CagA-positive *H. pylori* strains, these exosomes may potentially contribute to an increased risk of colorectal cancer [48] and decrease the risk of pancreatic cancer [49]. Exosomes derived from gastric cells infected with *H. pylori* also transfer the functional activated mesenchymal-epithelial transition factor that is internalized by tumor-associated macrophages [50]. After internalization, this factor seems to modulate tumor-associated macrophages to promote gastric cancer progression [50]. In the stomach, exosomes derived from the serum of *H. pylori*-infected patients suffering from chronic gastritis can induce the expression of soluble IL-6 receptor, which increases expression of the pro-inflammatory cytokine IL-1α in gastric epithelial cells [51]. Similarly, Shiga toxin (Stx) 2a secreted by enterohemorrhagic *E. coli* is captured by kidney cells, transported to recycling endosomes and released into extracellular milieu, where it could be either free or associated with exosomes [52]. In mice, exosome-associated Stx2a was found to induce more critical lethality and kidney damages than the free form of Stx2a [52]. Finally, cells intoxicated with the lethal toxin virulence factor secreted by *B. anthracis*, a toxin that cleaves most mitogen-activated protein kinase (MAPK) kinases (MAPKKs) and nucleotide binding oligomerization domain (NOD)-like receptor family pyrin domain containing 1 (NLRP1), leading to the inhibition of most MAPK signaling pathways and activation of NLRP1 inflammasomes, secrete exosomes delivering this toxin to naïve cells [53]. These exosomes deliver the lethal factor to naïve cells at a distance from the infection site, thus contributing to the diffusion and persistence of these toxins within the host [53].

### 2.2. Exosomes and Adaptive Immune Responses

Increasing evidence suggests that exosomes can modulate T and B cell differentiation, activation and proliferation by either directly interacting with B and T cells or indirectly via their capture by antigen-presenting cells (APCs) that present exosomal antigens to T cells, thus influencing adaptive immune responses. Indeed, it has been shown that exosomes secreted by dendritic cells and B lymphocytes express the major histocompatibility complex (MHC) class I and II loaded with antigens as well as CD86 and adhesion molecules on their surface [54]. In vitro, this exosomal complex can activate naïve or differentiated T lymphocytes [54]. Moreover, electronic microscopy analysis has revealed the presence of MHC-II molecules on the surface of follicular dendritic cells [55]. As these cells do not normally express MHC-II molecules, the latter probably come from the capture of exosomes secreted by adjacent B cells [55]. Similarly, dendritic cells infected with mycoplasma bacteria secrete exosomes that activate B cell proliferation without the help of T lymphocytes and in an antigen non-specific manner [33]. Tumor cells infected with *Mycoplasma* also secrete exosomes that activate B cells [33], which, in turn, secrete exosomes inhibiting T cell activation [56]. Because exosomes convey antigens and are enriched in MHC molecules, they may act as a direct or indirect antigen-presenting component.

#### 2.2.1. Direct Antigen Presentation

Exosomes can directly present their MHC-loaded antigens to T cells without the help of dendritic cells. However, the ability of exosomes derived from APCs to stimulate T cells is 10 to 20 times lower than that of APCs themselves [57,58]. Exosomes from dendritic cells carrying ovalbumin on MHC-I molecules activate CD8^+^ T hybridoma cells [59]. Dendritic cells infected with *M. tuberculosis* secrete exosomes carrying *M. tuberculosis* peptide-MHC-II-complexes that may activate T lymphocytes [60]. In addition, activated T cells are more susceptible to transcriptional modulation in response to exosomes derived from dendritic cells than resting T cells [61]. This exosomal stimulation results in an increased expression of inhibitory cytokines by T cells [61]. Exosomes from dendritic cells also induce the activation and proliferation of natural killer (NK) cells via the IL-15Rα receptor on the exosomal surface that binds to IL-15, and via the UL16 binding protein 1 (ULBP1), a ligand for the natural killer group 2 member D (NKG2D) receptor expressed by NK cells [62].

#### 2.2.2. Indirect Antigen Presentation

MHC-antigenic peptide complexes may be transported via exosomes to recipient APCs and, without the need of being processed by recipient cells, be used for the presentation of exogenous antigens to T lymphocytes and activation of these cells [63]. This indirect mode of antigen presentation is also called as MHC cross-dressing. Exosomes carrying antigens, in the presence of naïve dendritic cells, are able to activate T lymphocytes [64]. Although exosomes secreted by *M. tuberculosis-* or *M. bovis*- infected macrophages contain bacterial antigens as well as MHC and co-stimulatory molecules and can directly stimulate CD4^+^ and CD8^+^ T cells isolated from mycobacteria-sensitized mice, maximal stimulation of these cells requires the prior incubation of exosomes with APCs such as dendritic cells [22]. Exosomes derived from murine dendritic cells contain antigens and MHC-II molecules and induce the activation of T lymphocytes in recipient mice [58]. However, the activation of T lymphocytes in vitro by these exosomes requires the presence of dendritic cells, which constitute essential intermediaries for exosomal antigenic presentation [58]. Finally, it was reported that mast cell-derived exosomes are able to induce the maturation and functional activation of dendritic cells through the cross-presentation of antigens to T cells in vivo [65].

Activation of T cells leads to an increased number of secreted exosomes and to the release of a distinct exosomal population, depending on their activation status [66]. Exosomes derived from activated CD3^+^ T lymphocytes, together with IL-2, stimulate and induce the proliferation of resting CD3^+^ T lymphocytes [67]. Moreover, T cell cultures pulsed with exosomes purified from stimulated CD3⁺ T lymphocytes and IL-2 showed an increase in CD8^+^ T cells [67]. During cognate immune interaction, miRNAs are transferred unidirectional from T cells to APCs via exosomes in an antigen-dependent manner [68]. Exosomes derived from CD8^+^ T lymphocytes carry miR-150 and modulate macrophages, which in turn induce regulatory T (Treg) cells and inhibit effector T cell proliferation, thus suppressing specific immune responses [69]. Finally, Treg cells release a larger number of exosomes compared to other T lymphocytes. These exosomes may carry the CD39 and CD73 surface proteins, which induce the production of adenosine, a nucleotide that plays a major role in anti-inflammatory response and T cell suppression [70], and transport the miRNA let-7d that suppresses T helper (Th) 1 proliferation and cytokine secretion, thus mediating immunosuppression [71].

## 3. Exosomes and IBD

IBD, which comprises CD and UC, are a family of chronic immune-related disease of the gastrointestinal tract characterized by defects in intestinal homeostasis leading to chronic relapsing intestinal inflammation. The exact etiology of IBD is unclear but seems to result from an abnormal and prolonged inflammatory response to intestinal microbiota in genetically susceptible individuals [18]. Diagnosis of IBD currently relies on a combination of symptoms, biological, endoscopic, radiologic and histologic criteria and on their evolution over time [72], and available drugs only aim at controlling relapses, limiting recurrence and improving the quality of patients’ life but fail to definitively cure IBD [73]. The dysregulation of mucosal immunity plays a major role in IBD development and progression. The larger part of pro-inflammatory cytokines is secreted by CD4^+^ T cells, including Th1, Th17 and Th2. While elevated levels of Th1 cytokines, such as TNF-α, IL-12 and IFN-γ, and Th17 cytokines, such as IL-17, IL-21 and IL-22, were reported in the intestinal mucosa of CD patients, UC patients rather exhibit an increase in Th2 cytokines, such as IL-5 and IL-13, and Th17 cytokines [74].

It was reported that exosomes isolated from the colonic lumen of IBD patients contain higher amounts of IL-6, IL-8, IL-10 and TNF-α compared to exosomes from healthy subjects, and the levels of these pro-inflammatory molecules were positively correlated with CD severity score [75]. Moreover, exosomes from IBD patients induce the activation of colonic epithelial cells in vitro, which then produce a greater amount of IL-8 [75]. Exosomes from IBD patients and intestinal epithelial cells treated with these exosomes induce the migration of a higher number of macrophages in vitro than untreated intestinal epithelial cells [75]. Similarly, exosomes purified from the serum of mice treated with dextran sulfate sodium (DSS) to induce colitis contain 56 differentially expressed proteins compared to exosomes isolated from untreated mice and are able to activate the MAPK pathway and to induce the production of TNF-α in naïve macrophages [76]. Intestinal microbiota play a major role in the development of IBD [18]. Enteropathogenic bacteria, such as *Bacteroides fragilis*, secrete outer membrane vesicles that stimulate the release of intestinal mucosa-derived exosomes carrying an elevated level of C-C motif chemokine 20 (CCL20) and prostaglandin E2 (PGE2). CCL20 and PGE2 induce the recruitment and proliferation, respectively, of Th17 cells through the MyD88-mediated pathway, and cause inflammation [77]. The bacterial stimulation of human neutrophils induces the release of microvesicles that exert antibacterial effects in a manner that depends on β2 integrin function, continuous actin remodeling and glucose supply [78]. An increase in human proteins associated with antimicrobial defense, and more specifically to oxidative stress, was reported in extracellular vesicles (including exosomes, microvesicles and bacterial outer membrane vesicles) found on the interface between intestinal mucosa and intestinal lumen in IBD patients compared to control subjects [79]. In addition, an increase in proteins related to oxidative antimicrobial activity was shown in IBD patients and was correlated with an alteration in microbial functions [79]. Thus, the modification of the protein composition of extracellular vesicles may be associated with an alteration in microbial functions and may play a role in the abnormal interactions observed between the host and its microbiota in IBD patients [79].

**Figure 1:**
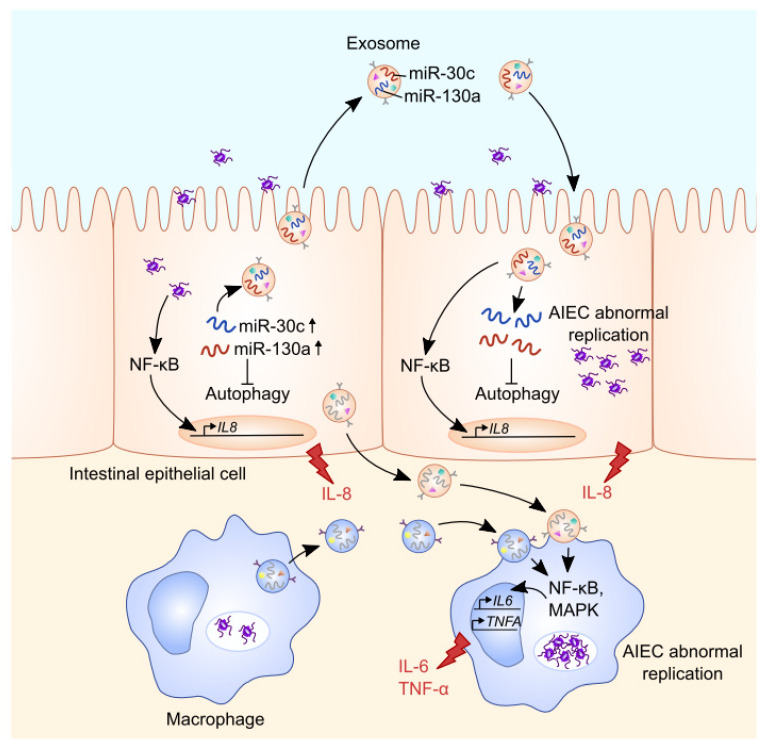
**Role of exosomes in intercellular communication during infection with Crohn disease (CD)-associated adherent-invasive *E. coli* (AIEC)**. AIEC infection leads to increased levels of miR-30c and miR-130a in intestinal epithelial cells (IECs), thereby inhibiting autophagy. These miRNAs can be transferred via the exosomal shuttle from cell to cell, impairing autophagy-mediated AIEC clearance and favoring AIEC colonization in intestinal epithelium (unpublished data). Moreover, the exosomes secreted by AIEC-infected IECs activate the NF-κB pathway in recipient IECs, enhancing IL-8 production. Similarly, increases in AIEC intracellular replication and the activation of NF-κB and MAPK pathways, leading to enhanced secretion of the pro-inflammatory cytokines IL-6 and TNF-α, were observed in macrophages receiving exosomes derived from IECs and macrophages infected with AIEC.

Exosomes purified from intestinal epithelial cells or macrophages infected with CD-associated AIEC can activate NF-κB and MAPK pathways in naïve macrophages, leading to an increased secretion of the pro-inflammatory cytokines IL-6 and TNF-α [20] (Figure 1). Exosomes derived from AIEC-infected intestinal epithelial cells can also activate the NF-κB pathway in uninfected intestinal epithelial cells, thereby increasing IL-8 secretion. Moreover, exosomes derived from AIEC-infected intestinal epithelial cells and macrophages promote AIEC replication inside exosome-receiving cells [20] (Figure 1). Our unpublished data showed that AIEC-infected intestinal epithelial cells secrete exosomes that have high levels of miR-30c and miR-130a. The exosomal miR-30c and miR-130a are then transferred via exosomes to recipient cells, where they target the mRNAs encoding the autophagy proteins ATG5 and ATG16L1, inhibiting ATG5 and ATG16L1 expression, respectively. This consequently leads to impaired autophagy-mediated clearance of intracellular AIEC, enhancing AIEC intracellular replication (Figure 1). The inhibition of these miRNAs in AIEC-infected cells abolishes the increase in miR-30c and miR-130a levels in exosomes secreted by these cells and in exosome-receiving cells, thus suppressing the inhibitory effect of exosomes on ATG5 and ATG16L1 expression and on autophagy-mediated AIEC clearance in exosome-receiving cells (unpublished data). In the ileal mucosa of CD patients, increased miR-30c and miR-130a levels and decreased *ATG5* and *ATG16L1* mRNA expression levels were observed compared to control subjects or UC patients [80], however, the levels of these miRNAs in CD patient-derived exosomes remain to be investigated. The infection of CEABAC10 transgenic mice expressing human carcinoembryonic antigen-related cell adhesion molecule (CEACAM) 6, a receptor expressed by intestinal epithelial cells for AIEC binding [81], with the AIEC reference strain LF82 induces the secretion of exosomes in intestinal lumen. Exosomes isolated from the luminal content of AIEC LF82-infected CEABAC10 transgenic mice trigger a pro-inflammatory response in naïve macrophages in vitro [20]. However, the exosomal components responsible for the induction of pro-inflammatory response in recipient cells remain to be identified. Together, these data highlight that exosomes are important mediators of host–AIEC interaction with their capacity to modulate innate immune responses.

## 4. Exosomes and Their Use as Prognostic and Therapeutic Tools

### 4.1. Exosomes as Promising Diagnostic Tools

Exosomes found in the biological fluids of patients with various diseases such as cancer, inflammatory diseases and infections exhibit modifications in their composition compared to those from healthy subjects. Thus, these vesicles represent an interesting tool for the identification of biomarkers using minimally- or non-invasive techniques [28].

The use of exosomes as biomarkers has been considered in the context of infectious diseases and especially in mycobacterial infection. Twenty mycobacterial proteins known to be involved in *M. tuberculosis* intracellular survival were identified in exosomes derived from the serum of *M. tuberculosis*-infected patients [82]. Moreover, the mRNA [83] and miRNA [25,84] profiles in exosomes derived from the serum differ between healthy subjects, patients with active tuberculosis and patients with latent tuberculosis and could therefore serve as biomarkers. Similarly, variation in the percentage of small RNA derived from repeated sequences of the genome in exosomes purified from the serum of tuberculosis patients may constitute a biomarker for the progression of this pathology [84]. Different levels of exosomal miR-148a-3p, miR-451a and miR-150-5p were also reported in pleural effusions of tuberculous lesions, making these miRNAs promising biomarkers for this disease [85]. Finally, a recent study suggested that a combination of the profiling of six differentially expressed miRNAs in plasma-derived exosomes and electronic health records may improve pulmonary tuberculosis and tuberculous meningitis diagnosis compared with miRNA or electronic health records alone [86].

**Figure 2:**
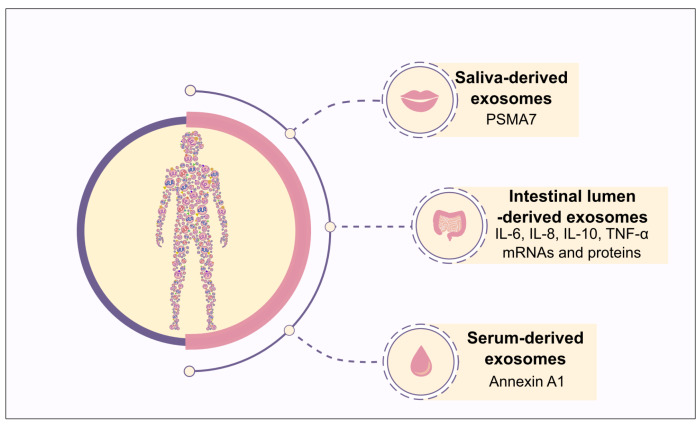
**Diagnostic potential of exosomes in IBD.** Exosomes derived from saliva, intestinal lumen as well as serum, which contain PSMA7, pro-inflammatory cytokine mRNAs and proteins, and annexin A1, respectively, could be used as promising biomarkers for IBD diagnosis.

The use of exosomes as biomarkers for non-cancerous inflammatory diseases such as rheumatoid arthritis, acute kidney failure, or IBD has also been investigated [87]. Concerning IBD (Figure 2), an increase in the level of exosomes containing annexin A1, a protein that stimulates intestinal mucosa wound repair in a murine model of colitis [88] and promotes the resolution of inflammation by binding to formyl peptide receptors expressed on immune [89] and epithelial [90] cells, was observed in the serum of IBD patients compared to healthy subjects and may be used as a potential biomarker of intestinal mucosa inflammation [15]. More than 2000 proteins were detected in exosomes purified from the saliva of IBD patients, a much larger amount than that found in healthy subjects [91]. Among these proteins, the most promising biomarker seems to be proteasome subunit alpha type 7 (PSMA7), which exhibits increased expression in IBD patients compared to healthy individuals [91]. This protein is involved in the ubiquitin–proteasome pathway and was previously reported to promote NOD1 degradation, thus limiting NOD1-mediated apoptosis, NF-κB activation and inflammation in the colon carcinoma cell line [92]. Exosomes from the colonic lumen of IBD patients contain significantly higher mRNA and protein levels of IL-6, IL-8, IL-10 and TNF-α compared with those from healthy individuals, and thus may contribute to IBD diagnosis [75].

### 4.2. Exosomes as Promising Therapeutic Tools

#### 4.2.1. Vaccination against Bacterial Infection with Exosomes

Exosomes can modulate immune responses and play a role as antigen presenters. Thus, their use as “vaccines” in infectious diseases has been suggested. A first study conducted in the context of bacterial infection reported that exosomes secreted by *M. bovis*- or *M. tuberculosis*- infected cells can activate antigen-specific CD4^+^ and CD8^+^ T cells in vivo and promote the activation and maturation of dendritic cells [22]. Macrophages treated with *M. tuberculosis* proteins release exosomes that recruit dendritic cells to the lungs [27] and activate dendritic cells, CD4^+^ and CD8^+^ T cells [23] as well as Th1 immune response protecting against *M. tuberculosis* infection [93] after intranasal administration into mice. In vivo, infection with *M. tuberculosis* leads to the secretion of exosomes that contribute to T cell responses, suggesting that the cross presentation of antigens may play a significant role in eliciting acquired immune responses during an infection with these bacteria [94].

Dendritic cells treated with diphtheria toxin secrete exosomes which, after intravenous injection into mice, stimulate a specific IgG response against this toxin [95]. Similarly, exosomes released by dendritic cells carry an antigen that exhibits a cross reactivity with the capsular polysaccharide 14 (Cps14) antigen of *Streptococcus pneumoniae* type 14. These exosomes stimulate the establishment of IgM and IgG responses against this antigen, thus protecting mice from an infection with *S. pneumoniae* type 14 [96].

#### 4.2.2. Therapeutic Potential of Exosomes in IBD

**Figure 3:**
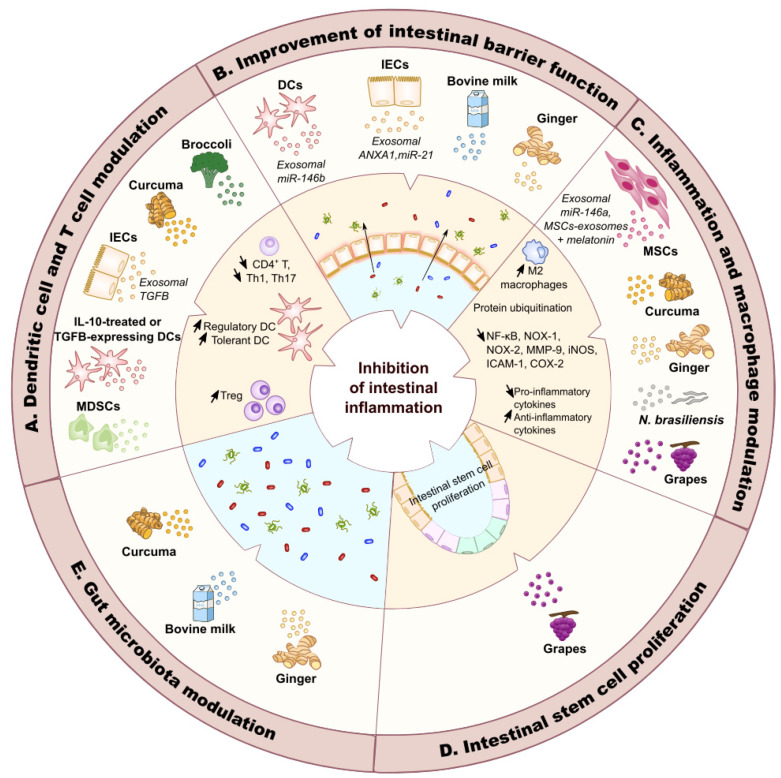
**Therapeutic potential of exosomes in IBD.** Exosomes derived from human cells, plants or worms have been shown to modulate various cell types and pathways to inhibit intestinal inflammation. A. Exosomes secreted by intestinal epithelial cells (IECs) [101], dendritic cells (DCs) [100] and myeloid-derived suppressor cells (MDSCs) [98] are able to limit the expansion of CD4^+^ T cells, and of Th1 and Th17 cells. These exosomes and exosomes derived from IL-10-treated DCs or TGF-β-expressing DCs and exosome-like nanoparticles from curcuma increase Treg cell development, while exosomes derived from IECs and exosome-like nanoparticles derived from curcuma favor regulatory DC expansion. Finally, broccoli-derived nanoparticles induce tolerant DCs [102]. Although the components responsible for the effects of exosome-like nanoparticles from curcuma or exosomes from MDSCs and DCs remain to be identified, the effects of exosomes derived from IECs seem to be mediated by exosomal TGF-β. B. Exosomes derived from DCs, IECs and ginger-derived exosome-like nanoparticles may improve intestinal barrier function. While specific ginger exosome-like nanoparticle component(s) involved in these effects have not yet been identified, it was shown that IEC-derived exosomes contain annexin A1 (ANXA1), a protein known to favor intestinal epithelial repair. Upon substance P stimulation, IECs also produce exosomes enriched in miR-21. These exosomes promote cell proliferation and migration, thus improving intestinal barrier function. C. Exosomes may have an impact on inflammation and macrophage modulation. Mesenchymal stem cell (MSC)-derived exosomes may limit colitis development by modulating protein ubiquitination and by increasing the number of anti-inflammatory M2 macrophages. MSC-derived exosomes that contain miR-146a and curcuma-derived exosome-like nanoparticles are able to inhibit NF-κB activation, thus limiting inflammation. In association with melatonin, MSC-derived exosomes also limit the activation of various pro-inflammatory pathways. Finally, exosome-like nanoparticles derived from ginger, curcuma and *N. brasiliensis* limit pro-inflammatory cytokine secretion and increase anti-inflammatory cytokine secretion. D. Exosome-like nanoparticles derived from grapes favor intestinal stem cell proliferation, thus limiting colitis development. E. Exosome-like nanoparticles derived from curcuma increase the abundance of butyrate-producing bacteria, while exosome-like nanoparticles from ginger are captured by *Lactobacillaceae* and modulate bacterial gene expression, thus increasing indole-3-carboxaldehyde expression that induces the production of IL-22 in the colon, leading to improved intestinal barrier function and decreased colitis development. NOX: NADPH oxidase; MMP-9: matrix metallopeptidase-9; iNOS: inducible nitric oxide synthase; ICAM-1: intercellular adhesion molecule-1; COX-2: cyclooxygenase-2.

Exosomes secreted by various cell types can modulate T lymphocyte activation and increase intestinal epithelial barrier function, thus constituting a new therapeutic strategy to reduce inflammation associated with IBD (Figure 3). Indeed, exosomes released by myeloid-derived suppressor cells (MDSC), a heterogeneous population of immature myeloid cells that regulates immune response and has been described in IBD [97], reduce the severity of DSS-induced colitis by inhibiting Th1 proliferation and promoting Treg cell expansion [98]. Similarly, exosomes secreted by dendritic cells treated with IL-10 inhibit colitis development in 2,4,6 trinitrobenzene sulfonic acid (TNBS)-treated rats by stimulating CD4^+^ CD25^+^ Treg cells [99]. In the same way, the administration of exosomes secreted by dendritic cells expressing TGF-β1 alleviates DSS-induced colitis, which is mediated by Th17 responses, by inducing CD4^+^ Foxp3^+^ Treg cell activation [100]. Intravenous injection of exosomes released by intestinal epithelial cells from untreated mice to DSS-treated mice reduces the severity of experimental colitis by decreasing the number of CD4^+^ T lymphocytes and by activating Treg cells and immunosuppressive dendritic cells, while the inhibition of exosome production in vivo increases the severity of DSS-induced colitis [101]. Moreover, exosomes secreted by intestinal epithelial cells after DSS treatment carry a higher amount of TGF-β1 and adhere to the epithelial cell adhesion molecule (EpCAM) expressed by intestinal epithelial cells to alleviate DSS-induced colitis [101].

Maintaining a functional intestinal barrier and epithelial restoration following injuries are also important factors to prevent intestinal inflammation (Figure 3). Exosomes derived from dendritic cells improve intestinal barrier function in a murine model of DSS-induced colitis by activating NF-κB via the exosomal miR-146b [44,103]. Annexin A1, which is overexpressed during pro-inflammatory response and plays a role in healing mucosal damages, is found in exosomes derived from intestinal epithelial cells [15]. The annexin A1-containing exosomes are able to activate mucosal healing in vitro and ex vivo [15]. Finally, it was recently shown that substance P, a neuropeptide, regulates the production of exosomes selectively enriched in miR-21 by intestinal epithelial cells [104]. In human colonocytes and mouse colonic crypts, these exosomes promote cell proliferation and migration, however, their effects on colitis remain to be investigated [104].

Several studies have reported that exosomes derived from mesenchymal stem cells (MSCs) represent a promising therapeutic tool (Figure 3). Indeed, the intraperitoneal administration of exosomes secreted by MSCs derived from bone marrow [105,106,107] or umbilical cord [108] to DSS-treated mice decreases disease activity score, weight loss, colon length shortening and histological score by increasing anti-inflammatory and reducing pro-inflammatory responses [105,106,107,108] more efficiently than MSCs alone [105,106,107]. It has been suggested that these exosomes may exert their effects on DSS-induced colitis by regulating protein ubiquitination [107] and by inducing the generation of immunosuppressive IL-10-producing M2 macrophages in the colon [108]. In vitro results confirmed that exosomes derived from MSCs inhibit inflammatory cytokine production by colonic macrophages stimulated with LPS and promote the polarization of these macrophages into M2 phenotype [108]. Moreover, MSC-derived exosomes alleviate colitis in vivo by inhibiting expression of IL-7 and inducible nitric oxide synthase (iNOS) in mouse colonic macrophages [105], thus limiting the production of nitric oxide and the expression of TNF-α, IL-6 and IL-1β and increasing IL-10 secretion [105,109]. MSC-derived extracellular vesicles, including exosomes and microparticles, also decrease the severity of TNBS-induced colitis in rats by suppressing activation of the NF-κB pathway [110], an effect probably mediated by the inhibition of TNF receptor associated factor 6 (TRAF6) and IL-1 receptor associated kinase 1 (IRAK1) by the anti-inflammatory miR-146a carried by these exosomes [111]. This suppression limits pro-inflammatory cytokine production and reduces nitric oxide production in colonic macrophages [110,111]. Finally, the injection of exosomes secreted by adipose tissue-derived MSCs combined with melatonin, an anti-inflammatory hormone, could limit inflammation caused by DSS-induced colitis in rat [112].

Some edible plants and food products, such as *Curcuma longa*, ginger, grapes, broccoli or bovine milk, contain exosomes and exosome-like nanoparticles that can relieve intestinal inflammation (Figure 3). Exosome-like nanoparticles carrying curcumin and derived from *Curcuma longa* limit DSS-induced colitis development in mice by suppressing NF-κB activation and by increasing Treg cell and regulatory dendritic cell expansion in the colonic mucosa [113]. Broccoli-derived nanoparticles protect mice from DSS-induced colitis by triggering the activation of adenosine monophosphate-activated protein kinase (AMPK) in dendritic cells, thus preventing dendritic cell activation and inducing tolerant dendritic cells [102]. Exosome-like nanoparticles purified from grape juice, when orally administrated to DSS-treated mice, are captured by LGR5^+^ intestinal stem cells and promote proliferation of these cells, thus enhancing epithelial tissue renewal and protecting mice from DSS-induced colitis [114]. Moreover, nanoparticles derived from grapefruit are taken up by intestinal macrophages and improve experimental colitis in mice by upregulating the expression of heme oxygenase-1 (HO-1) and inhibiting the production of IL-1β and TNF-α [115]. Mass spectrometry analysis of these grapefruit-derived exosomes revealed that they carry numerous proteins involved in the regulation of carbohydrates and lipid metabolism, however, their impact on macrophage physiology remains to be investigated [115]. Exosome-like nanoparticles derived from ginger are mostly captured by intestinal epithelial cells and macrophages [116]. In vivo, these vesicles alleviate DSS-induced colitis, promote intestinal healing by increasing survival and proliferation of intestinal epithelial cells, limit pro-inflammatory cytokine secretion and increase the release of anti-inflammatory cytokines, suggesting their potential in the prevention and treatment of IBD [116]. Finally, exosomes derived from bovine milk increase the levels of *Muc2*, *RegIIIγ*, *Myd88* and *Gata4* gene expression, as well as IgA and sIgA levels in the intestine, thus playing a role in maintaining the integrity of the mucus layer and intestinal barrier function in mice [117].

Nanoparticles derived from food products have also an impact on intestinal microbiota. Indeed, treatment with nanoparticle curcumin increases the abundance of butyrate-producing bacteria and fecal butyrate level in both the inflamed colon and normal colon in mice [113]. Similarly, bovine milk-derived nanoparticles alter gut microbiota composition and modulate their production of some short-chain fatty acids in murine gastrointestinal tract, enhancing epithelial barrier [117]. Finally, ginger-derived exosome-like nanoparticles are preferentially captured by *Lactobacillaceae* in a nanoparticle-lipid dependent manner and contain miRNAs that modulate bacterial gene expression [118]. Among them, ginger nanoparticle-derived mdo-miR7267-3p targets a monooxygenase (ycnE) of *Lactobacillus rhamnosus* (LGG), leading to the increased expression of indole-3-carboxaldehyde (I3A) [118]. I3A acts as a ligand for aryl hydrocarbon receptor and induces the production of IL-22 in the colon of DSS-treated mice, thus improving the maintenance of intestinal barrier function and decreasing the severity of experimental colitis [118]. These findings suggested that food product-derived nanoparticles may be used to target specific microbiota components and alleviate intestinal inflammation.

Extracellular vesicles derived from intestinal parasites could allow the development of new therapeutic strategies for IBD (Figure 3). Indeed, some intestinal parasites, and more particularly hookworms may have immunosuppressive properties and have been already used in clinical trials for IBD treatment [119,120]. Exosome-like extracellular vesicles released by the rodent intestinal parasite *Nippostrongylus brasiliensis* contain proteins and miRNAs potentially carrying immunomodulatory properties. Intraperitoneal injection of these vesicles to mice treated with TNBS decreases the level of the pro-inflammatory cytokines IL-6, IL-1β, IFN-γ and IL-17a and increases the level of the anti-inflammatory cytokine IL-10 in colonic tissue, thus preventing colitis development [121]. Parasite-derived extracellular vesicles could thus be used as a therapy for the treatment of IBD [121].

#### 4.2.3. Exosomes and Administration of Therapeutic Molecules

Exosomes are resistant to lysis by complement [122] and by RNases [5], which increases their stability in vivo by protecting their content. Therefore, the use of exosomes for the administration of therapeutic molecules has been considered [123]. Several strategies allow the incorporation of therapeutic molecules into exosomes, such as the direct modification of exosomal content by direct incubation, electroporation, transfection, ultrasound treatment, exosomal membrane permeabilization with saponins, sonication or freeze-thaw cycles [124]. To date, in the context of bacterial infection, only the antibacterial compound linezolid has been successfully incorporated in naïve exosomes by direct incubation of these vesicles with the molecule and has been shown to exert its effect in vivo [125]. The addition of a therapeutic molecule in exosomes can also be obtained by modifying the genome of exosome-donor cells to induce the expression and capture of the therapeutic compound in the secreted exosomes. This strategy has been successfully employed in the context of IBD. Exosomes secreted by BMDC genetically modified to express the gene encoding TGF-β1 (TGF-β/BMDC) were able to increase Treg cells and suppress Th17 response in DSS-treated mice, thus protecting against experimental colitis [100]. TGF-β was detected in exosomes released by TGF-β/BMDC but not in exosomes from non-modified BMDC. The intravenous administration of 10 µg of exosomes containing TGF-β to mice before DSS treatment helps to prevent weight loss, to reduce intestinal bleeding and disease activity index, while the administration of TGF-β alone does not confer any protection against DSS-induced colitis [100]. This suggested that the protective effects of exosomes on DSS-induced colitis could probably come from the increased stability of TGF-β in exosomes [100]. However, although able to delay the development of colitis, exosomes containing TGF-β did not inhibit the progression of colitis after disease onset [100].

## 5. Conclusions

A growing amount of evidence suggests that exosomes play a role in bacterial infection as well as in IBD progression, and a better understanding of the underlying mechanisms may provide new insights for de development of future diagnosis and therapeutic strategies. The presence of PAMPs and other immunomodulatory molecules in exosomes and the ability of these vesicles to modulate immune responses raise the potential of development of exosome-based “vaccines” for infectious diseases. Similarly, exosomes derived from different mammalian cell types, from parasites but also from edible plants, have exerted the potential to limit inflammation associated with IBD. Moreover, the stability of exosomes in various body fluids and the presence of some disease-associated components in these vesicles make the analysis of exosomal composition a promising strategy to identify minimally- or non-invasive biomarkers for diseases. Finally, the stability and packaging properties of exosomes point to their use as vectors of therapeutic molecules. However, further investigations are required to better understand the role of these vesicles in infection and IBD, their potential use as a diagnostic tool, to manage the incorporation of therapeutic molecules into these vesicles, to identify the most efficient targeting strategy and to evaluate the safety of these drug-loaded vesicles in vivo.

## References

[1] Carrière J., Barnich N., Nguyen H.T.T. (2016). Exosomes: From Functions in Host-Pathogen Interactions and Immunity to Diagnostic and Therapeutic Opportunities. Reviews of Physiology, Biochemistry and Pharmacology.

[2] Palmulli R., van Niel G. (2018). To be or not to be... secreted as exosomes, a balance finely tuned by the mechanisms of biogenesis. Essays Biochem..

[3] Subra C., Grand D., Laulagnier K., Stella A., Lambeau G., Paillasse M., De Medina P., Monsarrat B., Perret B., Silvente-Poirot S. (2010). Exosomes account for vesicle-mediated transcellular transport of activatable phospholipases and prostaglandins. J. Lipid Res..

[4] André F., Chaput N., Schartz N.E.C., Flament C., Aubert N., Bernard J., Lemonnier F., Raposo G., Escudier B., Hsu D.-H. (2004). Exosomes as Potent Cell-Free Peptide-Based Vaccine. I. Dendritic Cell-Derived Exosomes Transfer Functional MHC Class I/Peptide Complexes to Dendritic Cells. J. Immunol..

[5] Valadi H., Ekström K., Bossios A., Sjöstrand M., Lee J.J., Lötvall J.O. (2007). Exosome-mediated transfer of mRNAs and microRNAs is a novel mechanism of genetic exchange between cells. Nat. Cell Biol..

[6] Johnstone R.M., Adam M., Hammond J.R., Orr L., Turbide C. (1987). Vesicle formation during reticulocyte maturation. Association of plasma membrane activities with released vesicles (exosomes). J. Biol. Chem..

[7] Hessvik N.P., Øverbye A., Brech A., Torgersen M.L., Jakobsen I.S., Sandvig K., Llorente A. (2016). PIKfyve inhibition increases exosome release and induces secretory autophagy. Cell. Mol. Life Sci..

[8] Meldolesi J. (2018). Exosomes and Ectosomes in Intercellular Communication. Curr. Biol..

[9] McKelvey K.J., Powell K.L., Ashton A.W., Morris J.M., McCracken S.A. (2015). Exosomes: Mechanisms of Uptake. J. Circ. Biomarkers.

[10] Horibe S., Tanahashi T., Kawauchi S., Murakami Y., Rikitake Y. (2018). Mechanism of recipient cell-dependent differences in exosome uptake. BMC Cancer.

[11] Ying W., Riopel M., Bandyopadhyay G., Dong Y., Birmingham A., Seo J.B., Ofrecio J.M., Wollam J., Hernandez-Carretero A., Fu W. (2017). Adipose Tissue Macrophage-Derived Exosomal miRNAs Can Modulate In Vivo and In Vitro Insulin Sensitivity. Cell.

[12] Zhao H., Shang Q., Pan Z., Bai Y., Li Z., Zhang H., Zhang Q., Guo C., Zhang L., Wang Q. (2018). Exosomes From Adipose-Derived Stem Cells Attenuate Adipose Inflammation and Obesity Through Polarizing M2 Macrophages and Beiging in White Adipose Tissue. Diabetes.

[13] Herrera M.B., Fonsato V., Gatti S., Deregibus M.C., Sordi A., Cantarella D., Calogero R., Bussolati B., Tetta C., Camussi G. (2009). Human liver stem cell-derived microvesicles accelerate hepatic regeneration in hepatectomized rats. J. Cell. Mol. Med..

[14] Borges F.T., Melo S.A., Özdemir B.C., Kato N., Revuelta I., Miller C.A., Gattone V.H., LeBleu V.S., Kalluri R. (2013). TGF- β 1–Containing Exosomes from Injured Epithelial Cells Activate Fibroblasts to Initiate Tissue Regenerative Responses and Fibrosis. J. Am. Soc. Nephrol..

[15] Leoni G., Neumann P.-A., Kamaly N., Quiros M., Nishio H., Jones H.R., Sumagin R., Hilgarth R.S., Alam A., Fredman G. (2015). Annexin A1–containing extracellular vesicles and polymeric nanoparticles promote epithelial wound repair. J. Clin. Invest..

[16] Li D., Liu J., Guo B., Liang C., Dang L., Lu C., He X., Cheung H.Y.-S., Xu L., Lu C. (2016). Osteoclast-derived exosomal miR-214-3p inhibits osteoblastic bone formation. Nat. Commun..

[17] Arenaccio C., Federico M. (2017). The Multifaceted Functions of Exosomes in Health and Disease: An Overview. Exosomes in Cardiovascular Diseases.

[18] Ramos G.P., Papadakis K.A. (2019). Mechanisms of Disease: Inflammatory Bowel Diseases. Mayo Clin. Proc..

[19] Carrière J., Darfeuille-Michaud A., Nguyen H.T.T. (2014). Infectious etiopathogenesis of Crohn’s disease. World J. Gastroenterol..

[20] Carrière J., Bretin A., Darfeuille-Michaud A., Barnich N., Nguyen H.T.T. (2016). Exosomes Released from Cells Infected with Crohnʼs Disease–associated Adherent-Invasive Escherichia coli Activate Host Innate Immune Responses and Enhance Bacterial Intracellular Replication. Inflamm. Bowel Dis..

[21] Bhatnagar S., Shinagawa K., Castellino F.J., Schorey J.S. (2007). Exosomes released from macrophages infected with intracellular pathogens stimulate a proinflammatory response in vitro and in vivo. Blood.

[22] Giri P.K., Schorey J.S. (2008). Exosomes Derived from M. Bovis BCG Infected Macrophages Activate Antigen-Specific CD4+ and CD8+ T Cells In Vitro and In Vivo. PLoS ONE.

[23] Giri P.K., Kruh N.A., Dobos K.M., Schorey J.S. (2010). Proteomic analysis identifies highly antigenic proteins in exosomes from M. tuberculosis-infected and culture filtrate protein-treated macrophages. Proteomics.

[24] Singh P.P., Li L., Schorey J.S. (2015). Exosomal RNA from Mycobacterium tuberculosis -Infected Cells Is Functional in Recipient Macrophages. Traffic.

[25] Alipoor S.D., Mortaz E., Tabarsi P., Farnia P., Mirsaeidi M., Garssen J., Movassaghi M., Adcock I.M. (2017). Bovis Bacillus Calmette–Guerin (BCG) infection induces exosomal miRNA release by human macrophages. J. Transl. Med..

[26] Cheng Y., Schorey J.S. (2019). Extracellular vesicles deliver Mycobacterium RNA to promote host immunity and bacterial killing. EMBO Rep..

[27] Singh P.P., Smith V.L., Karakousis P.C., Schorey J.S. (2012). Exosomes Isolated from Mycobacteria-Infected Mice or Cultured Macrophages Can Recruit and Activate Immune Cells In Vitro and In Vivo. J. Immunol..

[28] Barile L., Vassalli G. (2017). Exosomes: Therapy delivery tools and biomarkers of diseases. Pharmacol. Ther..

[29] Afonso P.V., Janka-Junttila M., Lee Y.J., McCann C.P., Oliver C.M., Aamer K.A., Losert W., Cicerone M.T., Parent C.A. (2012). LTB4 Is a Signal-Relay Molecule during Neutrophil Chemotaxis. Dev. Cell.

[30] Majumdar R., Tavakoli Tameh A., Parent C.A. (2016). Exosomes Mediate LTB4 Release during Neutrophil Chemotaxis. PLoS Biol..

[31] Szatmary A.C., Nossal R., Parent C.A., Majumdar R. (2017). Modeling neutrophil migration in dynamic chemoattractant gradients: Assessing the role of exosomes during signal relay. Mol. Biol. Cell.

[32] Obregon C., Rothen-Rutishauser B., Gerber P., Gehr P., Nicod L.P. (2009). Active Uptake of Dendritic Cell-Derived Exovesicles by Epithelial Cells Induces the Release of Inflammatory Mediators through a TNF-α-Mediated Pathway. Am. J. Pathol..

[33] Quah B.J.C., O’Neill H.C. (2007). Mycoplasma contaminants present in exosome preparations induce polyclonal B cell responses. J. Leukoc. Biol..

[34] Wu Z., Li Y., Liu Q., Liu Y., Chen L., Zhao H., Guo H., Zhu K., Zhou N., Chai T.C. (2019). Pyroptosis engagement and bladder urothelial cell-derived exosomes recruit mast cells and induce barrier dysfunction of bladder urothelium after uropathogenic E. coli infection. Am. J. Physiol. Physiol..

[35] Diaz G., Wolfe L.M., Kruh-Garcia N.A., Dobos K.M. (2016). Changes in the Membrane-Associated Proteins of Exosomes Released from Human Macrophages after Mycobacterium tuberculosis Infection. Sci. Rep..

[36] Singh P.P., LeMaire C., Tan J.C., Zeng E., Schorey J.S. (2011). Exosomes Released from M.tuberculosis Infected Cells Can Suppress IFN-γ Mediated Activation of Naïve Macrophages. PLoS ONE.

[37] Wang J., Chen C., Xie P., Pan Y., Tan Y., Tang L. (2014). Proteomic analysis and immune properties of exosomes released by macrophages infected with Mycobacterium avium. Microbes Infect..

[38] Anand P.K., Anand E., Bleck C.K.E., Anes E., Griffiths G. (2010). Exosomal hsp70 induces a pro-inflammatory response to foreign particles including mycobacteria. PLoS ONE.

[39] Bhatnagar S., Schorey J.S. (2007). Exosomes Released from Infected Macrophages Contain Mycobacterium avium Glycopeptidolipids and Are Proinflammatory. J. Biol. Chem..

[40] Athman J.J., Wang Y., McDonald D.J., Boom W.H., Harding C.V., Wearsch P.A. (2015). Bacterial Membrane Vesicles Mediate the Release of Mycobacterium tuberculosis Lipoglycans and Lipoproteins from Infected Macrophages. J. Immunol..

[41] Alipoor S.D., Adcock I.M., Folkerts G., Garssen J., Mortaz E. (2019). A bioinformatics analysis of exosomal microRNAs released following mycobacterial infection. Int. J. Mycobacteriol..

[42] Yuan Q., Chen H., Yang Y., Fu Y., Yi Z. (2019). miR-18a promotes Mycobacterial survival in macrophages via inhibiting autophagy by down-regulation of ATM. J. Cell. Mol. Med..

[43] Fleming A., Sampey G., Chung M.-C., Bailey C., van Hoek M.L., Kashanchi F., Hakami R.M. (2014). The carrying pigeons of the cell: Exosomes and their role in infectious diseases caused by human pathogens. Pathog. Dis..

[44] Alexander M., Hu R., Runtsch M.C., Kagele D.A., Mosbruger T.L., Tolmachova T., Seabra M.C., Round J.L., Ward D.M., O’Connell R.M. (2015). Exosome-delivered microRNAs modulate the inflammatory response to endotoxin. Nat. Commun..

[45] Jiang K., Yang J., Guo S., Zhao G., Wu H., Deng G. (2019). Peripheral Circulating Exosome-Mediated Delivery of miR-155 as a Novel Mechanism for Acute Lung Inflammation. Mol. Ther..

[46] Wang J., Deng Z., Wang Z., Wu J., Gu T., Jiang Y., Li G. (2016). MicroRNA-155 in exosomes secreted from helicobacter pylori infection macrophages immunomodulates inflammatory response. Am. J. Transl. Res..

[47] Shimoda A., Ueda K., Nishiumi S., Murata-Kamiya N., Mukai S., Sawada S., Azuma T., Hatakeyama M., Akiyoshi K. (2016). Exosomes as nanocarriers for systemic delivery of the Helicobacter pylori virulence factor CagA. Sci. Rep..

[48] Strofilas A., Lagoudianakis E.E., Seretis C., Pappas A., Koronakis N., Keramidaris D., Koukoutsis I., Chrysikos I., Manouras I., Manouras A. (2012). Association of helicobacter pylori infection and colon cancer. J. Clin. Med. Res..

[49] Risch H.A., Lu L., Kidd M.S., Wang J., Zhang W., Ni Q., Gao Y.-T., Yu H. (2014). Helicobacter pylori Seropositivities and Risk of Pancreatic Carcinoma. Cancer Epidemiol. Biomarkers Prev..

[50] Che Y., Geng B., Xu Y., Miao X., Chen L., Mu X., Pan J., Zhang C., Zhao T., Wang C. (2018). Helicobacter pylori -induced exosomal MET educates tumour-associated macrophages to promote gastric cancer progression. J. Cell. Mol. Med..

[51] Chen Y., Wang X., Yu Y., Xiao Y., Huang J., Yao Z., Chen X., Zhou T., Li P., Xu C. (2018). Serum exosomes of chronic gastritis patients infected with Helicobacter pylori mediate IL-1α expression via IL-6 trans-signalling in gastric epithelial cells. Clin. Exp. Immunol..

[52] Watanabe-Takahashi M., Yamasaki S., Murata M., Kano F., Motoyama J., Yamate J., Omi J., Sato W., Ukai H., Shimasaki K. (2018). Exosome-associated Shiga toxin 2 is released from cells and causes severe toxicity in mice. Sci. Rep..

[53] Abrami L., Brandi L., Moayeri M., Brown M.J., Krantz B.A., Leppla S.H., van der Goot F.G. (2013). Hijacking Multivesicular Bodies Enables Long-Term and Exosome-Mediated Long-Distance Action of Anthrax Toxin. Cell Rep..

[54] Zeng F., Morelli A.E. (2018). Extracellular vesicle-mediated MHC cross-dressing in immune homeostasis, transplantation, infectious diseases, and cancer. Semin. Immunopathol..

[55] Denzer K., van Eijk M., Kleijmeer M.J., Jakobson E., de Groot C.J., Geuze H. (2000). Follicular Dendritic Cells Carry MHC Class II-Expressing Microvesicles at Their Surface. J. Immunol..

[56] Yang C., Chalasani G., Ng Y.-H., Robbins P.D. (2012). Exosomes Released from Mycoplasma Infected Tumor Cells Activate Inhibitory B Cells. PLoS ONE.

[57] Raposo G. (1996). B lymphocytes secrete antigen-presenting vesicles. J. Exp. Med..

[58] Théry C., Duban L., Segura E., Véron P., Lantz O., Amigorena S. (2002). Indirect activation of naïve CD4+ T cells by dendritic cell–derived exosomes. Nat. Immunol..

[59] Utsugi-Kobukai S., Fujimaki H., Hotta C., Nakazawa M., Minami M. (2003). MHC class I-mediated exogenous antigen presentation by exosomes secreted from immature and mature bone marrow derived dendritic cells. Immunol. Lett..

[60] Ramachandra L., Qu Y., Wang Y., Lewis C.J., Cobb B.A., Takatsu K., Boom W.H., Dubyak G.R., Harding C.V. (2010). Mycobacterium tuberculosis Synergizes with ATP To Induce Release of Microvesicles and Exosomes Containing Major Histocompatibility Complex Class II Molecules Capable of Antigen Presentation. Infect. Immun..

[61] Muller L., Mitsuhashi M., Simms P., Gooding W.E., Whiteside T.L. (2016). Tumor-derived exosomes regulate expression of immune function-related genes in human T cell subsets. Sci. Rep..

[62] Viaud S., Terme M., Flament C., Taieb J., André F., Novault S., Escudier B., Robert C., Caillat-Zucman S., Tursz T. (2009). Dendritic Cell-Derived Exosomes Promote Natural Killer Cell Activation and Proliferation: A Role for NKG2D Ligands and IL-15Rα. PLoS ONE.

[63] Campana S., De Pasquale C., Carrega P., Ferlazzo G., Bonaccorsi I. (2015). Cross-dressing: An alternative mechanism for antigen presentation. Immunol. Lett..

[64] Wolfers J., Lozier A., Raposo G., Regnault A., Théry C., Masurier C., Flament C., Pouzieux S., Faure F., Tursz T. (2001). Tumor-derived exosomes are a source of shared tumor rejection antigens for CTL cross-priming. Nat. Med..

[65] Skokos D., Botros H.G., Demeure C., Morin J., Peronet R., Birkenmeier G., Boudaly S., Mécheri S. (2003). Mast Cell-Derived Exosomes Induce Phenotypic and Functional Maturation of Dendritic Cells and Elicit Specific Immune Responses In Vivo. J. Immunol..

[66] van der Vlist E.J., Arkesteijn G.J.A., van de Lest C.H.A., Stoorvogel W., Nolte-’t Hoen E.N.M., Wauben M.H.M. (2012). CD4 + T cell activation promotes the differential release of distinct populations of nanosized vesicles. J. Extracell. Vesicles.

[67] Wahlgren J., Karlson T.D.L., Glader P., Telemo E., Valadi H. (2012). Activated Human T Cells Secrete Exosomes That Participate in IL-2 Mediated Immune Response Signaling. PLoS ONE.

[68] Mittelbrunn M., Gutiérrez-Vázquez C., Villarroya-Beltri C., González S., Sánchez-Cabo F., González M.Á., Bernad A., Sánchez-Madrid F. (2011). Unidirectional transfer of microRNA-loaded exosomes from T cells to antigen-presenting cells. Nat. Commun..

[69] Nazimek K., Ptak W., Nowak B., Ptak M., Askenase P.W., Bryniarski K. (2015). Macrophages play an essential role in antigen-specific immune suppression mediated by T CD8 + cell-derived exosomes. Immunology.

[70] Clayton A., Al-Taei S., Webber J., Mason M.D., Tabi Z. (2011). Cancer Exosomes Express CD39 and CD73, Which Suppress T Cells through Adenosine Production. J. Immunol..

[71] Okoye I.S., Coomes S.M., Pelly V.S., Czieso S., Papayannopoulos V., Tolmachova T., Seabra M.C., Wilson M.S. (2014). MicroRNA-Containing T-Regulatory-Cell-Derived Exosomes Suppress Pathogenic T Helper 1 Cells. Immunity.

[72] Tontini G.E. (2015). Differential diagnosis in inflammatory bowel disease colitis: State of the art and future perspectives. World J. Gastroenterol..

[73] Kim D.H., Cheon J.H. (2017). Pathogenesis of Inflammatory Bowel Disease and Recent Advances in Biologic Therapies. Immune Netw..

[74] Ahluwalia B., Moraes L., Magnusson M.K., Öhman L. (2018). Immunopathogenesis of inflammatory bowel disease and mechanisms of biological therapies. Scand. J. Gastroenterol..

[75] Mitsuhashi S., Feldbrügge L., Csizmadia E., Mitsuhashi M., Robson S.C., Moss A.C. (2016). Luminal Extracellular Vesicles (EVs) in Inflammatory Bowel Disease (IBD) Exhibit Proinflammatory Effects on Epithelial Cells and Macrophages. Inflamm. Bowel Dis..

[76] Wong W.-Y., Lee M.M.-L., Chan B.D., Kam R.K.-T., Zhang G., Lu A.-P., Tai W.C.-S. (2016). Proteomic profiling of dextran sulfate sodium induced acute ulcerative colitis mice serum exosomes and their immunomodulatory impact on macrophages. Proteomics.

[77] Deng Z., Mu J., Tseng M., Wattenberg B., Zhuang X., Egilmez N.K., Wang Q., Zhang L., Norris J., Guo H. (2015). Enterobacteria-secreted particles induce production of exosome-like S1P-containing particles by intestinal epithelium to drive Th17-mediated tumorigenesis. Nat. Commun..

[78] Timár C.I., Lőrincz Á.M., Csépányi-Kömi R., Vályi-Nagy A., Nagy G., Buzás E.I., Iványi Z., Kittel Á., Powell D.W., McLeish K.R. (2013). Antibacterial effect of microvesicles released from human neutrophilic granulocytes. Blood.

[79] Zhang X., Deeke S.A., Ning Z., Starr A.E., Butcher J., Li J., Mayne J., Cheng K., Liao B., Li L. (2018). Metaproteomics reveals associations between microbiome and intestinal extracellular vesicle proteins in pediatric inflammatory bowel disease. Nat. Commun..

[80] Nguyen H.T.T., Dalmasso G., Müller S., Carrière J., Seibold F., Darfeuille-Michaud A. (2014). Crohn’s disease-associated adherent invasive escherichia coli modulate levels of microRNAs in intestinal epithelial cells to reduce autophagy. Gastroenterology.

[81] Barnich N., Carvalho F.A., Glasser A., Darcha C., Jantscheff P., Allez M., Peeters H., Bommelaer G., Desreumaux P., Colombel J.-F. (2007). CEACAM6 acts as a receptor for adherent-invasive E. coli, supporting ileal mucosa colonization in Crohn disease. J. Clin. Investig..

[82] Kruh-Garcia N.A., Wolfe L.M., Chaisson L.H., Worodria W.O., Nahid P., Schorey J.S., Davis J.L., Dobos K.M. (2014). Detection of Mycobacterium tuberculosis peptides in the exosomes of patients with active and latent M. tuberculosis infection using MRM-MS. PLoS ONE.

[83] Lv L., Li C., Zhang X., Ding N., Cao T., Jia X., Wang J., Pan L., Jia H., Li Z. (2017). RNA Profiling Analysis of the Serum Exosomes Derived from Patients with Active and Latent Mycobacterium tuberculosis Infection. Front. Microbiol..

[84] Lyu L., Zhang X., Li C., Yang T., Wang J., Pan L., Jia H., Li Z., Sun Q., Yue L. (2019). Small RNA Profiles of Serum Exosomes Derived From Individuals With Latent and Active Tuberculosis. Front. Microbiol..

[85] Wang Y., Xu Y.-M., Zou Y.-Q., Lin J., Huang B., Liu J., Li J., Zhang J., Yang W.-M., Min Q.-H. (2017). Identification of differential expressed PE exosomal miRNA in lung adenocarcinoma, tuberculosis, and other benign lesions. Medicine (Baltimore)..

[86] Hu X., Liao S., Bai H., Wu L., Wang M., Wu Q., Zhou J., Jiao L., Chen X., Zhou Y. (2019). Integrating exosomal microRNAs and electronic health data improved tuberculosis diagnosis. EBioMedicine.

[87] Console L., Scalise M., Indiveri C. (2019). Exosomes in inflammation and role as biomarkers. Clin. Chim. Acta.

[88] Leoni G., Alam A., Neumann P.-A., Lambeth J.D., Cheng G., McCoy J., Hilgarth R.S., Kundu K., Murthy N., Kusters D. (2013). Annexin A1, formyl peptide receptor, and NOX1 orchestrate epithelial repair. J. Clin. Investig..

[89] Perretti M., D’Acquisto F. (2009). Annexin A1 and glucocorticoids as effectors of the resolution of inflammation. Nat. Rev. Immunol..

[90] Martin G.R., Perretti M., Flower R.J., Wallace J.L. (2008). Annexin-1 modulates repair of gastric mucosal injury. Am. J. Physiol. Liver Physiol..

[91] Zheng X., Chen F., Zhang Q., Liu Y., You P., Sun S., Lin J., Chen N. (2017). Salivary exosomal PSMA7: A promising biomarker of inflammatory bowel disease. Protein Cell.

[92] Yang L., Tang Z., Zhang H., Kou W., Lu Z., Li X., Li Q., Miao Z. (2013). PSMA7 Directly Interacts with NOD1 and Regulates its Function. Cell. Physiol. Biochem..

[93] Cheng Y., Schorey J.S. (2013). Exosomes carrying mycobacterial antigens can protect mice against Mycobacterium tuberculosis infection. Eur. J. Immunol..

[94] Smith V.L., Cheng Y., Bryant B.R., Schorey J.S. (2017). Exosomes function in antigen presentation during an in vivo Mycobacterium tuberculosis infection. Sci. Rep..

[95] Colino J., Snapper C.M. (2006). Exosomes from Bone Marrow Dendritic Cells Pulsed with Diphtheria Toxoid Preferentially Induce Type 1 Antigen-Specific IgG Responses in Naive Recipients in the Absence of Free Antigen. J. Immunol..

[96] Colino J., Snapper C.M. (2007). Dendritic cell-derived exosomes express a Streptococcus pneumoniae capsular polysaccharide type 14 cross-reactive antigen that induces protective immunoglobulin responses against pneumococcal infection in mice. Infect. Immun..

[97] Gabrilovich D.I., Nagaraj S. (2009). Myeloid-derived suppressor cells as regulators of the immune system. Nat. Rev. Immunol..

[98] Wang Y., Tian J., Tang X., Rui K., Tian X., Ma J., Ma B., Xu H., Lu L., Wang S. (2016). Exosomes released by granulocytic myeloid-derived suppressor cells attenuate DSS-induced colitis in mice. Oncotarget.

[99] Yang X., Meng S., Jiang H., Chen T., Wu W. (2010). Exosomes derived from interleukin-10-treated dendritic cells can inhibit trinitrobenzene sulfonic acid-induced rat colitis. Scand. J. Gastroenterol..

[100] Cai Z., Zhang W., Yang F., Yu L., Yu Z., Pan J., Wang L., Cao X., Wang J. (2012). Immunosuppressive exosomes from TGF-β1 gene-modified dendritic cells attenuate Th17-mediated inflammatory autoimmune disease by inducing regulatory T cells. Cell Res..

[101] Jiang L., Shen Y., Guo D., Yang D., Liu J., Fei X., Yang Y., Zhang B., Lin Z., Yang F. (2016). EpCAM-dependent extracellular vesicles from intestinal epithelial cells maintain intestinal tract immune balance. Nat. Commun..

[102] Deng Z., Rong Y., Teng Y., Mu J., Zhuang X., Tseng M., Samykutty A., Zhang L., Yan J., Miller D. (2017). Broccoli-Derived Nanoparticle Inhibits Mouse Colitis by Activating Dendritic Cell AMP-Activated Protein Kinase. Mol. Ther..

[103] Nata T., Fujiya M., Ueno N., Moriichi K., Konishi H., Tanabe H., Ohtake T., Ikuta K., Kohgo Y. (2013). MicroRNA-146b improves intestinal injury in mouse colitis by activating nuclear factor-κB and improving epithelial barrier function. J. Gene Med..

[104] Bakirtzi K., Man Law I.K., Fang K., Iliopoulos D., Pothoulakis C. (2019). MiR-21 in Substance P-induced exosomes promotes cell proliferation and migration in human colonic epithelial cells. Am. J. Physiol. Liver Physiol..

[105] Mao F., Wu Y., Tang X., Kang J., Zhang B., Yan Y., Qian H., Zhang X., Xu W. (2017). Exosomes Derived from Human Umbilical Cord Mesenchymal Stem Cells Relieve Inflammatory Bowel Disease in Mice. Biomed Res. Int..

[106] Ma Z.J., Wang Y.H., Li Z.G., Wang Y., Li B.Y., Kang H.Y., Wu X.Y. (2019). Immunosuppressive Effect of Exosomes from Mesenchymal Stromal Cells in Defined Medium on Experimental Colitis. Int. J. Stem Cells.

[107] Wu Y., Xu X., Kang J., Wang J., Wen Y., Yan Y., Qian H., Zhang X., Xu W., Mao F. (2018). Exosomes derived from human umbilical cord mesenchymal stem cells alleviate inflammatory bowel disease in mice through ubiquitination. Am. J. Transl. Res..

[108] Cao L., Xu H., Wang G., Liu M., Tian D., Yuan Z. (2019). Extracellular vesicles derived from bone marrow mesenchymal stem cells attenuate dextran sodium sulfate-induced ulcerative colitis by promoting M2 macrophage polarization. Int. Immunopharmacol..

[109] Bao C., Wang B., Yang F., Chen L. (2018). Blockade of Interleukin-7 Receptor Shapes Macrophage Alternative Activation and Promotes Functional Recovery After Spinal Cord Injury. Neuroscience.

[110] Yang J., Liu X.-X., Fan H., Tang Q., Shou Z.-X., Zuo D.-M., Zou Z., Xu M., Chen Q.-Y., Peng Y. (2015). Extracellular Vesicles Derived from Bone Marrow Mesenchymal Stem Cells Protect against Experimental Colitis via Attenuating Colon Inflammation, Oxidative Stress and Apoptosis. PLoS ONE.

[111] Wu H., Fan H., Shou Z., Xu M., Chen Q., Ai C., Dong Y., Liu Y., Nan Z., Wang Y. (2019). Extracellular vesicles containing miR-146a attenuate experimental colitis by targeting TRAF6 and IRAK1. Int. Immunopharmacol..

[112] Chang C., Chen C., Chiang J.Y., Sun C., Chen Y., Chen K. (2019). Synergistic effect of combined melatonin and adipose-derived mesenchymal stem cell (ADMSC )-derived exosomes on amelioration of dextran sulfate sodium ( DSS ) -induced acute colitis. Am. J. Transl. Res..

[113] Ohno M., Nishida A., Sugitani Y., Nishino K., Inatomi O., Sugimoto M., Kawahara M., Andoh A. (2017). Nanoparticle curcumin ameliorates experimental colitis via modulation of gut microbiota and induction of regulatory T cells. PLoS ONE.

[114] Ju S., Mu J., Dokland T., Zhuang X., Wang Q., Jiang H., Xiang X., Deng Z.B., Wang B., Zhang L. (2013). Grape exosome-like nanoparticles induce intestinal stem cells and protect mice from DSS-induced colitis. Mol. Ther..

[115] Wang B., Zhuang X., Deng Z.-B., Jiang H., Mu J., Wang Q., Xiang X., Guo H., Zhang L., Dryden G. (2014). Targeted Drug Delivery to Intestinal Macrophages by Bioactive Nanovesicles Released from Grapefruit. Mol. Ther..

[116] Zhang M., Viennois E., Prasad M., Zhang Y., Wang L., Zhang Z., Han M.K., Xiao B., Xu C., Srinivasan S. (2016). Edible ginger-derived nanoparticles: A novel therapeutic approach for the prevention and treatment of inflammatory bowel disease and colitis-associated cancer. Biomaterials.

[117] Tong L., Hao H., Zhang X., Zhang Z., Lv Y., Zhang L., Yi H. (2020). Oral Administration of Bovine Milk-Derived Extracellular Vesicles Alters the Gut Microbiota and Enhances Intestinal Immunity in Mice. Mol. Nutr. Food Res..

[118] Teng Y., Ren Y., Sayed M., Hu X., Lei C., Kumar A., Hutchins E., Mu J., Deng Z., Luo C. (2018). Plant-Derived Exosomal MicroRNAs Shape the Gut Microbiota. Cell Host Microbe.

[119] Summers R.W., Elliott D.E., Urban J.F., Thompson R.A., Weinstock J.V. (2005). Trichuris suis therapy for active ulcerative colitis: A randomized controlled trial. Gastroenterology.

[120] Croese J. (2006). A proof of concept study establishing Necator americanus in Crohn’s patients and reservoir donors. Gut.

[121] Eichenberger R.M., Ryan S., Jones L., Buitrago G., Polster R., Montes de Oca M., Zuvelek J., Giacomin P.R., Dent L.A., Engwerda C.R. (2018). Hookworm Secreted Extracellular Vesicles Interact With Host Cells and Prevent Inducible Colitis in Mice. Front. Immunol..

[122] Clayton A., Harris C.L., Court J., Mason M.D., Morgan B.P. (2003). Antigen-presenting cell exosomes are protected from complement-mediated lysis by expression of CD55 and CD59. Eur. J. Immunol..

[123] Bunggulawa E.J., Wang W., Yin T., Wang N., Durkan C., Wang Y., Wang G. (2018). Recent advancements in the use of exosomes as drug delivery systems. J. Nanobiotechnol..

[124] Antimisiaris S.G., Mourtas S., Marazioti A. (2018). Exosomes and exosome-inspired vesicles for targeted drug delivery. Pharmaceutics.

[125] Yang X., Shi G., Guo J., Wang C., He Y. (2018). Exosome-encapsulated antibiotic against intracellular infections of methicillin-resistant Staphylococcus aureus. Int. J. Nanomed..

